# Astrocytes Modulate the Polarization of CD4^+^ T Cells to Th1 Cells

**DOI:** 10.1371/journal.pone.0086257

**Published:** 2014-01-28

**Authors:** Eléonore Beurel, Laurie E. Harrington, William Buchser, Vance Lemmon, Richard S. Jope

**Affiliations:** 1 Departments of Psychiatry and Behavioral Sciences, and Biochemistry and Molecular Biology, Miller School of Medicine, University of Miami, Miami, Florida, United States of America; 2 Department of Cell Biology, University of Alabama at Birmingham, Birmingham, Alabama, United States of America; 3 Department of Genetics, Washington University, St Louis, Missouri, United States of America; 4 Miami Project to Cure Paralysis, University of Miami, Miami, Florida, United States of America; University of California, Riverside, United States of America

## Abstract

T-cell characteristics are dynamic and influenced by multiple factors. To test whether cells and the environment in the central nervous system (CNS) can influence T-cells, we tested if culturing mouse CD4^+^ T-cells on mouse primary astrocytes, compared with standard feeder cells, modified T-cell polarization to Th1 and Treg subtypes. Astrocytes supported the production of Th1 cells and Tregs, which was diminished by inflammatory activation of astrocytes, and glutamate accumulation that may result from impaired glutamate uptake by astrocytes strongly promoted Th1 production. These results demonstrate that astrocytes and the environment in the CNS have the capacity to regulate T-cell characteristics.

## Introduction

Recently there has been a rapidly increasing recognition that extensive interactions occur between the immune and nervous systems [1], [2]. This progress is revising previous dogmas about the insular actions of these two systems, revealing instead that there are often bidirectional immune-neural interactions. An important one of these is the actions of T cells in the central nervous system (CNS), which is now known to include both beneficial and detrimental influences of T cells on CNS functions [3], [4], [5]. Beneficial CNS actions of T cells have been particularly well-established for their roles in contributing to cognition [6], [7], [8], [9], [10] and hippocampal neurogenesis in adult mammals [5], [7], [11]. Also well-established are the detrimental actions of T cells in certain CNS diseases, such as being major drivers of the onset and progression of multiple sclerosis [12], [13].

Multiple sclerosis is the most common inflammatory demyelinating disease of the CNS and is widely considered an autoimmune disease caused by autoreactive T cells [13], [14]. Several of the clinical, immunological, and neuropathological features of MS are modeled in experimental autoimmune encephalomyelitis (EAE), which is induced in susceptible mice by eliciting an immune response to injected myelin antigens [15], [16]. The two major populations of effector T helper (Th) cells present in the CNS of mice that are thought to contribute to EAE are interferon-γ (IFNγ)-producing Th1 cells and interleukin-17A (IL-17A)-producing Th17 cells. The differentiation of naive CD4^+^ T cells into subtypes results from the activation of their T cell receptor (TCR) and co-stimulatory molecules in the presence of specific cytokines produced by the innate immune system [17]. IFNγ and IL-12 induce the differentiation of CD4^+^ T cells to Th1 cells [18], [19], whereas TGFβ induces anti-inflammatory regulatory T (Treg) cell production [20]. Recent discoveries that T cell subtype characteristics can be dynamic [21], [22] have added a layer of complexity and indicates that environmental influences are capable of modulating the subtype characteristics of T cells.

Although it is evident that T cells in the CNS have a variety of actions, little is known about how the environment within the CNS affects T cells. Astrocytes are situated close to blood vessels, thus being an early cellular contact of infiltrating CD4^+^ T cells [23], [24]. Using in vitro co-cultures of cells, previous studies have reported that microglia and astrocytes, as well as neurons, can influence the priming or activation of T cells [25], [26], [27], [28], [29], [30]. However it is not clear if astrocytes can affect T cell differentiation characteristics, even though astrocytes are capable of producing key regulatory cytokines [23]. In the present study, the co-culture approach was applied to test if mouse primary astrocytes are capable of influencing the differentiation of co-cultured CD4^+^ T cells to Th1 cells or Tregs.

## Materials and Methods

### Ethics Statement

All mice were housed and treated in accordance with National Institutes of Health guidelines and procedures with mice were approved by the University of Miami Institutional Animal Care and Use Committee (11-233, 11-238).

### Mice

C57BL/6 (6–8 weeks) mice were purchased from the Jackson Laboratories. Mice were housed in a light and temperature controlled room and treated in accordance with NIH and University of Miami Institutional Animal Care and Use Committee regulations.

### Astrocyte culture

Primary glia were prepared from cerebral cortices of 1 day old C57Bl/6 mice as described [31], [32], and cultured in DMEM//F12 medium supplemented with 10% fetal bovine serum (FBS), 0.3% glucose, 2 mM L-glutamine, 10 U/mL penicillin and 10 µg/mL streptomycin. For separation of astrocytes and microglia, after 10 days of culture the cells were shaken (30 h; 250 rpm) and released microglia were discarded, to obtain >99% pure astrocytes as determined by immunostaining with the astrocyte marker glial fibrillary acidic protein (GFAP) (Millipore, Bedford, MA), <1% were microglia. Protein-free E. coli (K235) LPS was prepared as described [33]. Cells were left untreated or stimulated with 100 ng/mL LPS for 6 h (to induce cytokine production) in medium supplemented with 10% FBS.

### CD4^+^ T cell isolation and activation

Spleens and lymph nodes were collected and single-cell suspensions were prepared by mechanical disruption in RPMI 1640 medium supplemented with 10% FBS, 100 IU/mL of penicillin, 100 µg/mL of streptomycin, 1× nonessential amino acids, 1 µM sodium pyruvate, 2.5 µM β-mercaptoethanol and 2 mM L-glutamine (R-10). CD4^+^ T cells were isolated by magnetic sorting with DynaLbeads mouse CD4^+^ beads according to the manufacturer's instructions (Invitrogen). Methods for differentiation of T cells were adapted from previously described procedures [34]. Conventional irradiated (3,000 rads) spleen and draining lymph node feeder cells were used as APCs, except where indicated otherwise, and were cultured with purified CD4^+^ T cells at a ratio of 5∶1. Alternatively, the same number of CD4^+^ T cells was cultivated on a confluent monolayer of astrocytes. CD4^+^ T cells were activated with 2.5 µg/mL anti-CD3 (clone 145-11), Th1 cells were differentiated by the addition of recombinant IL-12 (10 ng/mL; R&D Systems) and anti-IL-4 (10 µg/mL; clone 11B11), and Tregs were prepared by incubation with TGFβ (5 ng/mL), anti-IL-4 (10 µg/mL; clone 11B11) and anti-IFN-γ (10 µg/mL; clone XMG1.2).

CD4^+^ T cells were cultivated with astrocytes in R10 medium. Where indicated, cells were treated with 10 ng/mL brain-derived nerve growth factor (BDNF), 100 ng/mL nerve growth factor (NGF), 10 ng/mL glial derived nerve factor (GDNF), 100 µM glutamate, or 100 µM MK-801 (Sigma).

### Intracellular cytokine staining

Astrocytes and CD4+ T cells were collected by mechanical pipetting and were stimulated for 4 h with PMA (50 ng/mL; Alexis) and ionomycin (750 ng/mL; Sigma) in the presence of Brefeldin A (BFA) at the recommended concentrations (BD Pharmingen) in a 96 well plate. Standard intracellular cytokine staining was carried out as described [34]. Cells were first stained extracellularly with APC–conjugated anti-CD4 (eBioscience) and fixed and permeabilized with Cytofix/Cytoperm solution (BD Pharmingen) and then were stained intracellularly with eFluo450-conjugated anti-IFN-γ, phycoerythrin-conjugated anti-IL-17A, (eBioscience). Samples were acquired on a LSRII (BD) and data were analyzed with FlowJo software (Tree Star, Inc.).

### Carboxyfluorescein succinimidyl ester (CFSE) labeling

CD4^+^ T cells were suspended at a density of 10^7^ cells per mL in PBS. CFSE (Molecular Probe) diluted in PBS was added to an equal volume of prewarmed cell suspension at a final concentration of 5 µM and the suspension was mixed rapidly. Cells were incubated at room temperature for 7 min and the reaction was stopped with FBS. Cells were centrifuged and resuspended in culture medium.

### Microscopic evaluation of the number of CD4^+^ T cells per astrocyte

To determine if the astrocytes and the CD4^+^ T cells come in close contact, the proximity of the nuclei of CD4^+^ T cells to the nuclei of astrocytes was evaluated by microscopy. The method was modified from [35]. Cells were co-cultured, stained with Hoechst and fixed with 3.2% PFA. A ThermoFisher Cellomics ArrayScan VTI automated fluorescent microscope was used to image the wells with a 10× objective at 1024×1024 resolution with 9 fields per well of a 24-well plate containing co-cultured astrocytes and CD4^+^ T cells. Hoechst was used to segment and identify the nuclei of all cells, and the size and intensity of each individual nucleus was identified using Cellomic's “TARGET ACTIVATION” algorithm. This technique is comparable to looking at forward scatter and Hoechst MFI in flow-cytometry, only the location information about astrocytes and T cells is preserved. Spotfire DecisionSite software (TIBCO) was used to analyze the data to distinguish between the two populations of cells, in the same way that populations of cells are ‘gated’ in flow cytometry (based on controls with only one of the cell-types present).

### Statistical analysis

Statistical significance was analyzed with a Mann-Whitney test or one way ANOVA with Bonferroni post-test using Prism software. A p value of less than 0.05 was considered significant.

## Results

### Astrocytes are sufficient to induce differentiation of CD4^+^ T cells to Th1 cells

**Figure 1:**
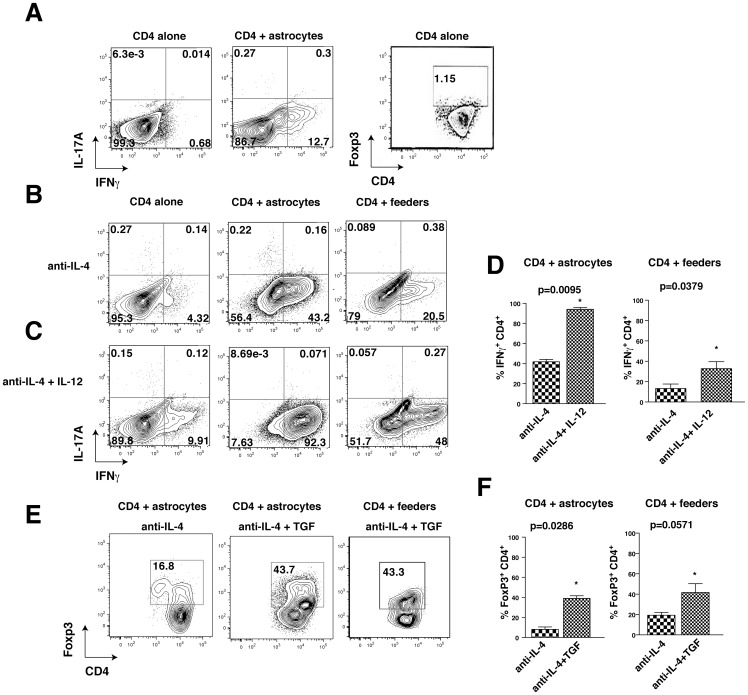
Astrocytes alone are sufficient to induce Th1 cell production. (A) CD4^+^ T cells were cultivated for 4 days on no feeder cells or on a monolayer of astrocytes. (B) CD4^+^ T cells were cultivated for 4 days on no feeder cells, on a monolayer of astrocytes, or on irradiated splenocyte feeder cells with 10 µg/mL anti-IL-4. (C) CD4^+^ T cells were cultivated for 4 days on no feeder cells, on a monolayer of astrocytes, or on irradiated splenocyte feeder cells with 10 µg/mL anti-IL-4 and 10 ng/mL IL-12. (A–D) IFNγ-producing, Foxp3 expressing and IL-17A-producing CD4^+^ T cells were analyzed by flow cytometry after restimulation with ionomycin/PMA/BFA for 4 h. Flow cytometry analyses were quantified and expressed as mean ± s.e.m. (for the data in D: n = 4–8, Mann-Whitney test, *p<0.05 compared with cells incubated without anti-IL-12). (E, F) CD4^+^ T cells were cultivated for 4 days on a monolayer of astrocytes or on irradiated splenocyte feeder cells with 10 µg/mL anti-IL-4, with or without 5 ng/mL TGFβ. Foxp3-expressing cells were analyzed by flow cytometry. Flow cytometry analyses were quantified and expressed as mean ± s.e.m. (n = 4, Mann-Whitney test, *p<0.05 compared with cells incubated without TGFβ).

We tested if astrocytes influence the polarization of CD4^+^ T cells to Th1 or Treg cells by comparing T cell populations after cultivating CD4^+^ T cells for 4 days on a monolayer of mouse primary astrocytes or on conventional irradiated spleen and draining lymph node feeder cells. Culturing CD4^+^ T cells with astrocytes without supplements of additional cytokines was sufficient to induce polarization of 12.7% of cells to IFNγ-producing CD4^+^ T cells, a marker of Th1 cells (Figure 1A). Addition of anti-IL-4 antibody (Figure 1B) increased the IFNγ-producing CD4^+^ T cell population by 3.4-fold to 42±2% of cells (quantitation in Figure 1C), whereas culturing CD4^+^ T cells on conventional feeder cells with anti-IL-4 only polarized 13.2±4% of CD4^+^ T cells to IFNγ-producing cells (Figure 1C). IL-12 is classically used to polarize CD4^+^ T cells to IFNγ-producing cells. Upon addition of IL-12 (Figure 1C, D), astrocytes promoted the differentiation of 94±2% CD4^+^ T cells towards Th1 cells , whereas IL-12 addition to feeder cell cultures resulted in the production of only 33±7% of IFNγ-producing CD4**^+^** T cells. These results demonstrate that astrocytes can be potent inducers of Th1 cell production, and that the effects of astrocytes are additive to the effect of IL-12 on promoting Th1 cell differentiation.

Culturing CD4^+^ T cells on astrocytes also supported polarization to Tregs. Culturing CD4^+^ T cells on astrocytes with added TGFβ and anti-IL-4 strongly induced Treg cell (39±2%) production to an extent equivalent to cells cultured on conventional feeder cells (42±9%) (Figure 1E, F).

**Figure 2:**
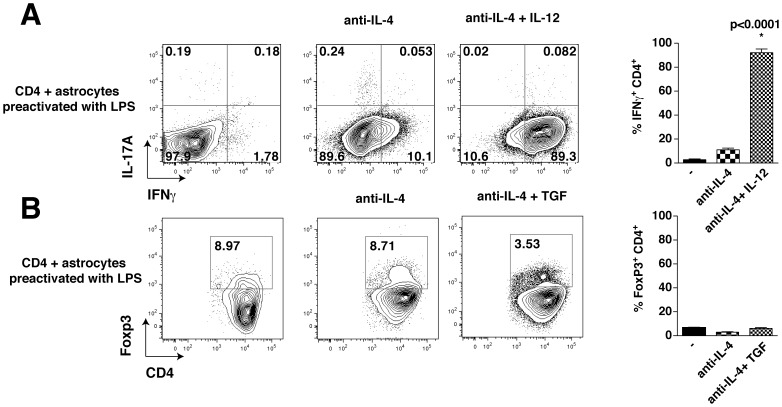
Preactivation of astrocytes reduces Th1 differentiation. CD4^+^ T cells were cultivated for 4 days on a monolayer of astrocytes that were pre-activated with LPS for 18 h, with or without 10 µg/mL anti-IL-4 and 10 ng/mL IL-12 where indicated. IFNγ-producing (A) and Foxp3 expressing (B) CD4^+^ T cells were analyzed by flow cytometry after restimulation with ionomycin/PMA/BFA for 4 h. Flow cytometry analyses were quantified and expressed as mean ± s.e.m. (for the data in A: n = 3, one-way ANOVA test, *p<0.05; for the data in B: n = 2–4).

Inflammation usually coincides with increased infiltration of T cells in the CNS in conditions such as multiple sclerosis and EAE. Therefore, we tested if activated astrocytes that produce increased amounts of cytokines influence Th differentiation differently from resting astrocytes. Astrocytes were pre-activated by overnight stimulation with lipopolysaccharide (LPS), followed by washing the cells before co-culturing with CD4^+^ T cells. Surprisingly, activated astrocytes inhibited Th1 differentiation in the absence of IL-12, but were without effect in the presence of IL-12 (Figure 2A). Activated astrocytes also inhibited Treg differentiation (Figure 2B).

These results indicate that resting and activated astrocytes differently modulate T cells. Without activation astrocytes promote the production of Th1 and Treg cells, whereas this is impaired upon culturing CD4^+^ T cells on pre-activated astrocytes.

### Cross-talk between Th1 cells and astrocytes is necessary to promote full Th1 differentiation

**Figure 3:**
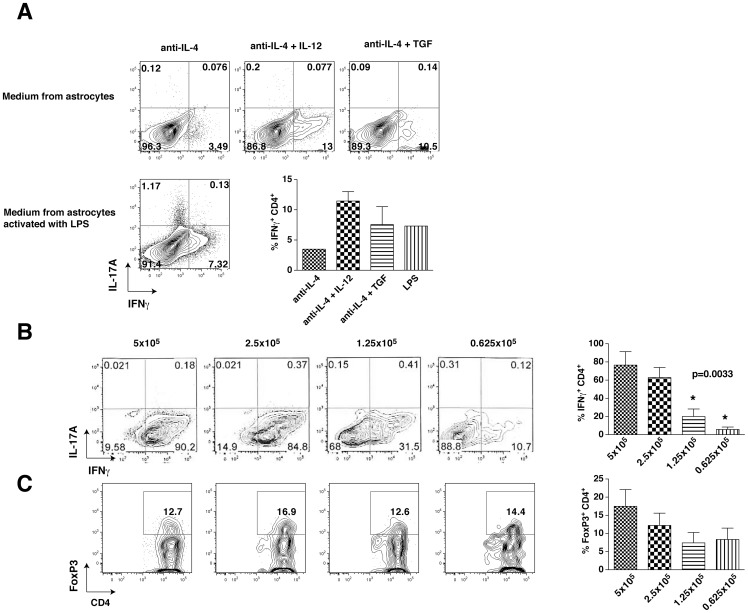
Cross-talk between Th1 cells and astrocytes is necessary to promote maximal Th1 differentiation. (A) CD4^+^ T cells were polarized toward Th1 cells for 4 days in conditioned medium from unstimulated or LPS-stimulated astrocytes supplemented with 10 ng/mL IL-12 (Th1) or 5 ng/mL TGFβ (Treg). Cells were washed and stimulated for 4 h with PMA/ionomycin/BFA and IFNγ-producing CD4^+^ T and Foxp3 expressing CD4^+^ T cells were analyzed by flow cytometry. Flow cytometry analyses were quantified and expressed as mean ± s.e.m. (n = 2). (B–C) Different quantities of CD4^+^ T cells were cultivated on an astrocyte monolayer and polarized toward (B) Th1, or (C) Treg cells for 4 days in medium supplemented with 10 ng/mL IL-12 (Th1), or 5 ng/mL TGFβ (Treg). Cells were washed, stimulated for 4 h with PMA/ionomycin/BFA, and IFNγ-producing and Foxp3-expressing CD4^+^ T cells were analyzed by flow cytometry. Flow cytometry analyses were quantified and expressed as mean ± s.e.m. (n = 3, , one-way ANOVA test, *p<0.05 compared to 5×10^5^ cells).

To test the hypothesis that a factor secreted by astrocytes promoted Th1 differentiation, CD4^+^ T cells were cultivated with conditioned medium from cultured astrocytes. There was no effect of the conditioned medium on Th1 cell differentiation (Figure 3A), since it did not induce Th1 differentiation (Figure 1). This suggests that it is not a factor secreted by astrocytes in the absence of CD4^+^ T cells that promotes Th1 cell production. Instead, we hypothesized that CD4^+^ T cells stimulate astrocytes to release a factor that promotes Th1 differentiation. To test this, serial dilutions of CD4^+^ T cells were cultivated with astrocytes and the proportion of the generation of IFNγ-producing cells was measured. Increasing dilutions of CD4^+^ T cells cultivated with astrocytes led to decreased percentages of Th1 cell production (Figure 3B), while there was no difference for Treg differentiation (Figure 3C). This suggests that there is a feedback loop by which the CD4^+^ T cells signal to astrocytes to induce production of factors that promote Th1 cell differentiation.

### Are mitogenic factors involved in Th1 cell differentiation induced in the presence of astrocytes?

**Figure 4:**
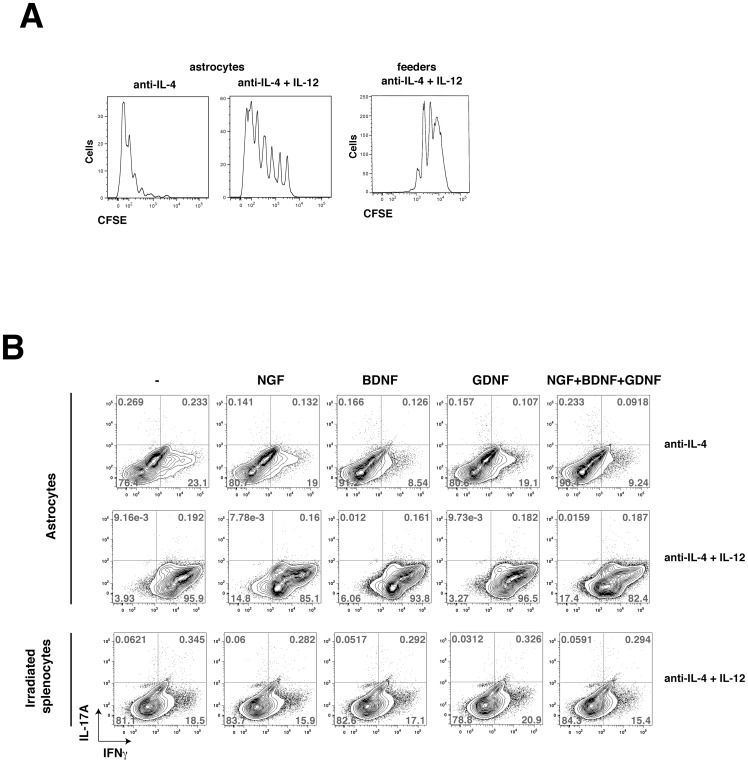
Th1 differentiation is not dependent on GDNF, BDNF or NGF. (A) CD4^+^ T cells were polarized toward Th1 cells for 3 days cultured on astrocytes or with irradiated feeder cells in medium supplemented with 10 ng/mL IL-12 (Th1). Cells were washed and stimulated for 4 hr with PMA/ionomycin and CD4^+^ T cells were analyzed for CFSE staining by flow cytometry gated on IFNγ^+^CD4^+^ T cells. Data are representative of 2 independent experiments. The rate of cell division was higher in cells incubated in Th1-polarizing conditions than undifferentiated cells. (B) CD4^+^ T cells cultivated on astrocytes or irradiated splenocytes were stimulated with 10 ng/mL GDNF, 10 ng/mL BDNF, 100 ng/mL NGF, or a combination as indicated, in the presence or not of Th1 polarizing conditions for 4 days. Cells were washed and stimulated with PMA/ionomycin/BFA for 4 h and IFNγ-producing cells were analyzed by flow cytometry. Plots are representative of 2 independent experiments.

CFSE staining of CD4**^+^** T cells cultured on astrocytes showed that cells incubated in Th1 polarizing conditions had a higher rate of cell division than undifferentiated CD4^+^ T cells or than cells incubated on irradiated lymph node cells and splenocytes in Th1 polarizing conditions (Figure 4A), suggesting that astrocytes may produce a mitogenic factor that promotes CD4**^+^** T cell proliferation and differentiation. We tested if the addition of several mitogenic factors known to be secreted by astrocytes [36], [37] modulate Th1 differentiation of CD4^+^ cells cultured on splenocytes, including nerve growth factor (NGF), glial derived nerve factor (GDNF) and brain-derived nerve growth factor (BDNF). However, there was no increase in the Th1 cell differentiation in the presence of these factors (Figure 4B).

### Glutamate modulates Th1 cell production

**Figure 5:**
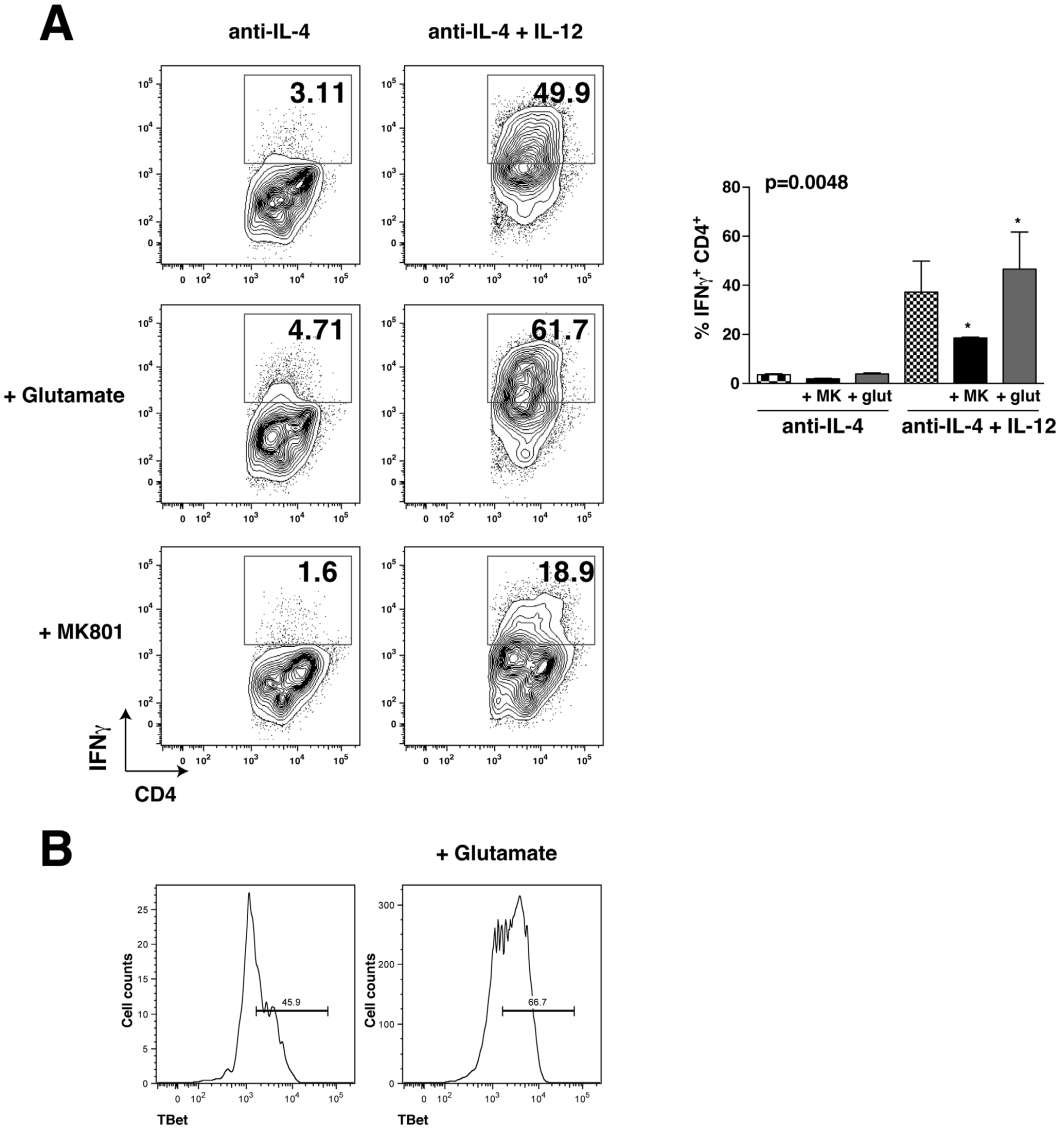
Th1 cell differentiation is promoted by glutamate. (A) CD4^+^ T cells were cultivated on irradiated splenocytes in the presence of 10 µg/mL anti-IL-4 alone or with 10 ng/mL IL-12 for 4 days, with or without 100 µM MK801 or 100 µM glutamate, each of which was reloaded after 2 days of polarization. Cells were washed and restimulated with PMA/ionomycin/BFA for 4 h and analyzed by flow cytometry. Flow cytometry analyses were quantified and expressed as mean ± s.e.m. (n = 3, one-way ANOVA test, *p<0.05). (B) CD4^+^ T cells were cultivated on irradiated splenocytes in the presence 10 µg/mL anti-IL-4 for 4 days with or without 100 µM glutamate, and cells were washed and restimulated with PMA/ionomycin/BFA for 4 h and analyzed by flow cytometry. The histogram represents TBet expressing cells.

An important function of astrocytes is the buffering of glutamate, which is critical for controlling extracellular glutamate levels and for protecting neurons from excitotoxicity [36], [37]. Since this function can be impaired in CNS diseases, resulting in increased extracellular glutamate levels, we tested if glutamate impacts Th1 cell differentiation. Addition of glutamate promoted Th1 differentiation of CD4**^+^** T cells cultured on splenocytes in the presence of anti-IL-4 and IL-12 (Figure 5). Conversely, treatment with the selective NMDA receptor antagonist MK801 reduced Th1 cell differentiation (Figure 5A). Moreover, addition of glutamate on CD4**^+^** T cells was sufficient to increase TBet expression, a marker of Th1 differentiation (Figure 5B). This suggests that in addition to producing cytokines critical for Th1 cell differentiation, the regulation of glutamate levels by astrocytes may also be an important modulator of the production of Th1 cells, similar to a previous report that glutamate released from DCs can regulate the secretion of Th1 cytokines [38].

### Th1 cells are dependent on CD4 for their differentiation on astrocytes

**Figure 6:**
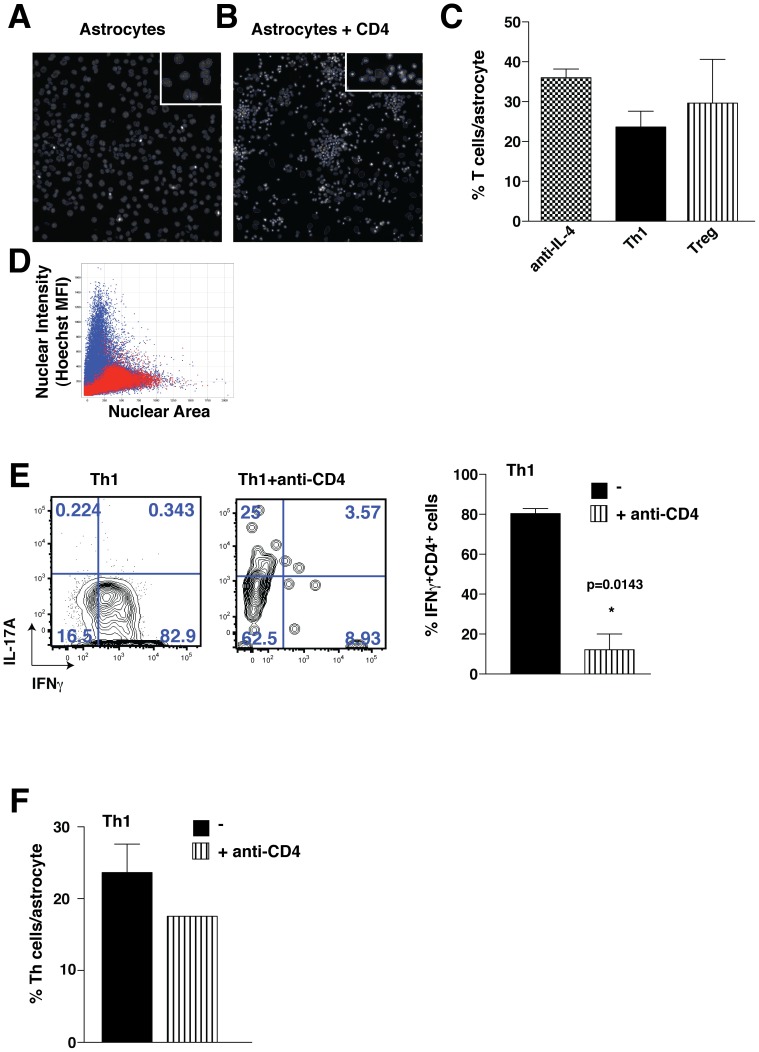
Th1 cell differentiation is promoted by CD4^+^ T cell activation. CD4^+^ T cells were cultivated on astrocytes in the presence of 10 µg/mL anti-IL-4 alone (anti-IL-4), with 10 µg/mL anti-IL-4 and 10 ng/mL IL-12 (Th1), or with 10 µg/mL anti-IL-4 and 2.5 ng/mL TGFβ (Treg) for 4 days. Cells were stained with Hoechst and fixed and analyzed using an ArrayScan VTI automated fluorescent microscope. (A) astrocytes alone with nuclei traced (a 20-fold magnification is shown in the small box), (B) astrocytes co-cultured with CD4^+^ T cells with nuclei traced (a 20-fold magnification is shown in the small box), (C) quantitation of percent of CD4^+^ T cells associated per astrocyte (mean ± s.e.m.; n = 3), (D) scatter plot showing the cells identified as astrocytes (red) and CD4^+^ T cells (blue). (E) CD4^+^ T cells were cultivated on astrocytes in the presence of 10 µg/mL anti-IL-4 and 10 ng/mL IL-12 for 4 days, with or without 10 µg/mL anti-CD4 (GK1.5). Cells were washed, and restimulated with PMA/ionomycin/BFA for 4 h and analyzed by flow cytometry. The histogram represents IFNγ-expressing CD4^+^ T cells (n = 2, unpaired t-test *p<0.05). (F) CD4^+^ T cells were cultivated on astrocytes in the presence of 10 µg/mL anti-IL-4 and with 10 ng/mL IL-12 for 4 days, with or without 10 µg/mL anti-CD4 (GK1.5). Cells were stained with Hoechst and fixed and analyzed with an ArrayScan VTI automated fluorescent microscope. Bars represent mean ± s.e.m. (n = 3).

Because the extent of direct interaction between CD4^+^ T cells and feeder cells may have a regulatory role in the differentiation of CD4^+^ T cells towards specific lineages, we examined if the number of CD4^+^ T cells associated with astrocytes was different among subpopulations of Th cells by measuring the number of CD4^+^ T cells associated with astrocytes using high content analysis. Astrocytes (Figure 6A, D) and T cells (Figure 6B,D) were distinguished based on nuclear size and intensity, based on thresholds established from control wells with a single cell type. CD4^+^ T cells were observed to be in close proximity to astrocytes (Figure 6B), and there were similar numbers of astrocyte-associated anti-IL-4 treated CD4^+^ T cells, Th1, and Tregs (Figure 6C). We next tested if blocking the CD4-mediated cell-cell interaction influences the differentiation of Th1 cells on astrocytes by incubation with a neutralizing antibody for CD4. Blocking CD4 was sufficient to block the differentiation towards Th1 cultured on astrocytes (Figure 6E), without affecting the percent of CD4^+^ T cells associated per astrocyte (Figure 6F), suggesting that the CD4 signaling is important for Th1 differentiation of CD4^+^ T cells cultured on astrocytes.

## Discussion

In contrast to previous dogmas that T cell subtypes and characteristics are stable, it is rapidly becoming clear that T cell characteristics are dynamic and subject to change in response to the environment and other factors [21], [22]. This raises the likelihood that T cells in the CNS are responsive to alterations in the environment created by resident cells. To begin to test if astrocytes can modify T cell characteristics, we used the straightforward paradigm of testing in vitro if astrocytes are capable of influencing the development of CD4^+^ T cells to different subtypes. The results show that resting astrocytes predominantly promote the development of Th1 cells, but this is diminished after activation of astrocytes.

During MS and EAE, peripheral Th1 cells accumulate in the CNS and contribute to demyelination, whereas Tregs are protective [13], [14]. Here we provide evidence that CNS resident astrocytes may also play a role in contributing to the regulation of Th1 and Treg cells. Astrocytes promoted Th1 differentiation in the absence or presence of exogenous IL-12, but this was impaired when CD4^+^ T cells were cultured on astrocytes pre-activated with LPS. This raises the possibility that depending on the environment in the CNS, astrocytes can contribute to the orientation of T cell characteristics towards subsets of Th cells. Although T cells in the CNS are thought to predominantly express subtype characteristics prior to infiltration, these results suggest that astrocytes may contribute to shifts in the subtype characteristics, or influence the development of any immature infiltrated CD4^+^ T cells, although each of these will require in vivo investigations.

In order to determine which specific astrocytic factors contribute to their modulation of Th1 cell differentiation, we analyzed candidates known to be involved in the functions of astrocytes. Among their actions, astrocytes are involved in neurotransmitter regulation, have mitogenic functions, and are modulators of inflammation [36], [37]. Because the proliferation of Th1 cells was increased in the presence of astrocytes compared with cells cultured on irradiated splenocytes, we tested if mitogenic factors secreted by astrocytes may be involved. However, incubation with NGF, BNDF or GDNF did not modulate Th1 cell differentiation, indicating that astrocytes can regulate Th1 cells by other factors or at the MHC level. The latter is consistent with the previous proposal that the strength of the TCR-MHC interaction is critical for Th1 differentiation and proliferation.

Glutamate appears to be an important regulator of T cell function. T cells have been shown to express many of the subtypes of glutamate receptors [39], [40], [41], [42]. Exposure to glutamate has been reported to modulate several functions and the proliferation of T cells by activating glutamate receptors expressed by T cells [38], [43], [44], [45], [46], [47], [48], [49]. Furthermore, the NMDA receptor antagonist memantine was reported to promote the development of Tregs [50]. A major function of astrocytes is to take up the neurotransmitter glutamate to prevent its accumulation in the synapse where it can reach levels sufficient to cause excitotoxicity in some neurological diseases. We found that glutamate has the capacity to regulate Th1 cells, as addition of exogenous glutamate promoted the production of Th1 cells. Thus, although it has been known for several years that astrocytes can interact with T cells via MHC-TCR interactions, these findings indicate that astrocytes also may affect the characteristics of T cells in the CNS, such as Th1 cell production, but whether this is critical in the development of EAE or other diseases remains to be determined. Furthermore, CD4 activation was found to be important for the differentiation of CD4^+^ T cells toward Th1 cells cultured on astrocytes, confirming that the TCR pathway plays an important role, since CD4 is a co-receptor of the TCR.

Although it is evident that T cells in the CNS have a variety of actions, evidence that the environment within the CNS affects resident T cells is only recently being identified. Thus, significant advances have recently been made concerning mechanisms regulating the infiltration of T cells and their actions in the CNS, but still to be defined is the dynamicity of their characteristics and the influence of the environment in the CNS on their characteristics and actions. The recent rapid growth of identified actions of T cells in healthy and diseased CNS and of the dynamic characteristics of T cells indicates that the capacity for unanticipated CNS-immune interactions should not be underestimated, such as the capacity of CNS cells to influence T cell characteristics as reported here.

## References

[1] Hanisch UK, Johnson TV, Kipnis J (2008) Toll-like receptors: roles in neuroprotection? Trends Neurosci 31: 176–182. 18329736 10.1016/j.tins.2008.01.005

[2] Ron-Harel N, Cardon M, Schwartz M (2011) Brain homeostasis is maintained by “danger” signals stimulating a supportive immune response within the brain's borders. Brain Behav Immun 25: 1036–1043. 21182929 10.1016/j.bbi.2010.12.011

[3] Schwartz M, Moalem G, Leibowitz-Amit R, Cohen IR (1999) Innate and adaptive immune responses can be beneficial for CNS repair. Trends Neurosci 22: 295–299. 10370250 10.1016/s0166-2236(99)01405-8

[4] Schwartz M, Shechter R (2010) Systemic inflammatory cells fight off neurodegenerative disease. Nat Rev Neurol 6: 405–410. 20531383 10.1038/nrneurol.2010.71

[5] Ziv Y, Schwartz M (2008) Orchestrating brain-cell renewal: the role of immune cells in adult neurogenesis in health and disease. Trends Mol Med 14: 471–478. 18929506 10.1016/j.molmed.2008.09.004

[6] Kipnis J, Cohen H, Cardon M, Ziv Y, Schwartz M (2004) T cell deficiency leads to cognitive dysfunction: implications for therapeutic vaccination for schizophrenia and other psychiatric conditions. Proc Natl Acad Sci U S A 101: 8180–8185. 15141078 10.1073/pnas.0402268101 PMC419577

[7] Ziv Y, Ron N, Butovsky O, Landa G, Sudai E, et al (2006) Immune cells contribute to the maintenance of neurogenesis and spatial learning abilities in adulthood. Nat Neurosci 9: 268–275. 16415867 10.1038/nn1629

[8] Brynskikh A, Warren T, Zhu J, Kipnis J (2008) Adaptive immunity affects learning behavior in mice. Brain Behav Immun 22: 861–869. 18249087 10.1016/j.bbi.2007.12.008

[9] Ron-Harel N, Segev Y, Lewitus GM, Cardon M, Ziv Y, et al (2008) Age-dependent spatial memory loss can be partially restored by immune activation. Rejuvenation Res 11: 903–913. 18803478 10.1089/rej.2008.0755

[10] Kipnis J, Derecki NC, Yang C, Scrable H (2008) Immunity and cognition: what do age-related dementia, HIV-dementia and ‘chemo-brain’ have in common? Trends Immunol 29: 455–463. 18789764 10.1016/j.it.2008.07.007

[11] Wolf SA, Steiner B, Akpinarli A, Kammertoens T, Nassenstein C, et al (2009) CD4-positive T lymphocytes provide a neuroimmunological link in the control of adult hippocampal neurogenesis. J Immunol 182: 3979–3984. 19299695 10.4049/jimmunol.0801218

[12] Goverman J (2009) Autoimmune T cell responses in the central nervous system. Nat Rev Immunol 9: 393–407. 19444307 10.1038/nri2550 PMC2813731

[13] Ontaneda D, Hyland M, Cohen JA (2012) Multiple sclerosis: new insights in pathogenesis and novel therapeutics. Annu Rev Med 63: 389–404. 21888515 10.1146/annurev-med-042910-135833

[14] Hafler DA (2004) Multiple sclerosis. J Clin Invest 113: 788–794. 15067307 10.1172/JCI21357 PMC362131

[15] Constantinescu CS, Farooqi N, O'Brien K, Gran B (2011) Experimental autoimmune encephalomyelitis (EAE) as a model for multiple sclerosis (MS). Br J Pharmacol 164: 1079–1106. 21371012 10.1111/j.1476-5381.2011.01302.x PMC3229753

[16] Stuve O, Kieseier BC, Hemmer B, Hartung HP, Awad A, et al (2010) Translational research in neurology and neuroscience 2010: multiple sclerosis. Arch Neurol 67: 1307–1315. 20625066 10.1001/archneurol.2010.158 PMC3670826

[17] Zhu J, Yamane H, Paul WE (2010) Differentiation of effector CD4 T cell populations (*). Annu Rev Immunol 28: 445–489. 20192806 10.1146/annurev-immunol-030409-101212 PMC3502616

[18] Hsieh CS, Macatonia SE, Tripp CS, Wolf SF, O'Garra A, et al (1993) Development of TH1 CD4+ T cells through IL-12 produced by Listeria-induced macrophages. Science 260: 547–549. 8097338 10.1126/science.8097338

[19] Frasca D, Adorini L, Landolfo S, Doria G (1985) Enhancing effect of IFN-gamma on helper T cell activity and IL 2 production. J Immunol 134: 3907–3911. 3157753

[20] Fu S, Zhang N, Yopp AC, Chen D, Mao M, et al (2004) TGF-beta induces Foxp3 + T-regulatory cells from CD4 + CD25 − precursors. Am J Transplant 4: 1614–1627. 15367216 10.1111/j.1600-6143.2004.00566.x

[21] Hirota K, Duarte JH, Veldhoen M, Hornsby E, Li Y, et al (2011) Fate mapping of IL-17-producing T cells in inflammatory responses. Nat Immunol 12: 255–263. 21278737 10.1038/ni.1993 PMC3040235

[22] Mukasa R, Balasubramani A, Lee YK, Whitley SK, Weaver BT, et al (2010) Epigenetic instability of cytokine and transcription factor gene loci underlies plasticity of the T helper 17 cell lineage. Immunity 32: 616–627. 20471290 10.1016/j.immuni.2010.04.016 PMC3129685

[23] Nair A, Frederick TJ, Miller SD (2008) Astrocytes in multiple sclerosis: a product of their environment. Cell Mol Life Sci 65: 2702–2720. 18516496 10.1007/s00018-008-8059-5 PMC2858316

[24] Ransohoff RM, Brown MA (2012) Innate immunity in the central nervous system. J Clin Invest 122: 1164–1171. 22466658 10.1172/JCI58644 PMC3314450

[25] Aloisi F, Ria F, Penna G, Adorini L (1998) Microglia are more efficient than astrocytes in antigen processing and in Th1 but not Th2 cell activation. J Immunol 160: 4671–4680. 9590212

[26] Aloisi F (1999) The role of microglia and astrocytes in CNS immune surveillance and immunopathology. Adv Exp Med Biol 468: 123–133. 10635024 10.1007/978-1-4615-4685-6_10

[27] Aloisi F, Ria F, Columba-Cabezas S, Hess H, Penna G, et al (1999) Relative efficiency of microglia, astrocytes, dendritic cells and B cells in naive CD4+ T cell priming and Th1/Th2 cell restimulation. Eur J Immunol 29: 2705–2714. 10508245 10.1002/(SICI)1521-4141(199909)29:09<2705::AID-IMMU2705>3.0.CO;2-1

[28] Becher B, Bechmann I, Greter M (2006) Antigen presentation in autoimmunity and CNS inflammation: how T lymphocytes recognize the brain. J Mol Med (Berl) 84: 532–543. 16773356 10.1007/s00109-006-0065-1

[29] Liu Y, Teige I, Birnir B, Issazadeh-Navikas S (2006) Neuron-mediated generation of regulatory T cells from encephalitogenic T cells suppresses EAE. Nat Med 12: 518–525. 16633347 10.1038/nm1402

[30] Nikcevich KM, Gordon KB, Tan L, Hurst SD, Kroepfl JF, et al (1997) IFN-gamma-activated primary murine astrocytes express B7 costimulatory molecules and prime naive antigen-specific T cells. J Immunol 158: 614–621. 8992975

[31] McCarthy KD, de Vellis J (1980) Preparation of separate astroglial and oligodendroglial cell cultures from rat cerebral tissue. J Cell Biol 85: 890–902. 6248568 10.1083/jcb.85.3.890 PMC2111442

[32] Beurel E, Jope RS (2008) Differential regulation of STAT family members by glycogen synthase kinase-3. J Biol Chem 283: 21934–21944. 18550525 10.1074/jbc.M802481200 PMC2494932

[33] Hirschfeld M, Ma Y, Weis JH, Vogel SN, Weis JJ (2000) Cutting edge: repurification of lipopolysaccharide eliminates signaling through both human and murine toll-like receptor 2. J Immunol 165: 618–622. 10878331 10.4049/jimmunol.165.2.618

[34] Harrington LE, Hatton RD, Mangan PR, Turner H, Murphy TL, et al (2005) Interleukin 17-producing CD4+ effector T cells develop via a lineage distinct from the T helper type 1 and 2 lineages. Nat Immunol 6: 1123–1132. 16200070 10.1038/ni1254

[35] Buchser WJ, Laskow TC, Pavlik PJ, Lin HM, Lotze MT (2012) Cell-mediated autophagy promotes cancer cell survival. Cancer Res 72: 2970–2979. 22505650 10.1158/0008-5472.CAN-11-3396 PMC3505669

[36] Allaman I, Belanger M, Magistretti PJ (2011) Astrocyte-neuron metabolic relationships: for better and for worse. Trends Neurosci 34: 76–87. 21236501 10.1016/j.tins.2010.12.001

[37] Fiacco TA, Agulhon C, McCarthy KD (2009) Sorting out astrocyte physiology from pharmacology. Annu Rev Pharmacol Toxicol 49: 151–174. 18834310 10.1146/annurev.pharmtox.011008.145602

[38] Pacheco R, Oliva H, Martinez-Navio JM, Climent N, Ciruela F, et al (2006) Glutamate released by dendritic cells as a novel modulator of T cell activation. J Immunol 177: 6695–6704. 17082582 10.4049/jimmunol.177.10.6695

[39] Levite M (2001) Nervous immunity: neurotransmitters, extracellular K+ and T-cell function. Trends Immunol 22: 2–5. 11286675 10.1016/s1471-4906(00)01799-3

[40] Lombardi G, Dianzani C, Miglio G, Canonico PL, Fantozzi R (2001) Characterization of ionotropic glutamate receptors in human lymphocytes. Br J Pharmacol 133: 936–944. 11454668 10.1038/sj.bjp.0704134 PMC1572842

[41] Pacheco R, Gallart T, Lluis C, Franco R (2007) Role of glutamate on T-cell mediated immunity. J Neuroimmunol 185: 9–19. 17303252 10.1016/j.jneuroim.2007.01.003

[42] Pacheco R, Riquelme E, Kalergis AM (2010) Emerging evidence for the role of neurotransmitters in the modulation of T cell responses to cognate ligands. Cent Nerv Syst Agents Med Chem 10: 65–83. 20236043 10.2174/187152410790780154

[43] Ganor Y, Besser M, Ben-Zakay N, Unger T, Levite M (2003) Human T cells express a functional ionotropic glutamate receptor GluR3, and glutamate by itself triggers integrin-mediated adhesion to laminin and fibronectin and chemotactic migration. J Immunol 170: 4362–4372. 12682273 10.4049/jimmunol.170.8.4362

[44] Pacheco R, Ciruela F, Casado V, Mallol J, Gallart T, et al (2004) Group I metabotropic glutamate receptors mediate a dual role of glutamate in T cell activation. J Biol Chem 279: 33352–33358. 15184389 10.1074/jbc.M401761200

[45] Boldyrev AA, Carpenter DO, Johnson P (2005) Emerging evidence for a similar role of glutamate receptors in the nervous and immune systems. J Neurochem 95: 913–918. 16271044 10.1111/j.1471-4159.2005.03456.x

[46] Poulopoulou C, Markakis I, Davaki P, Nikolaou C, Poulopoulos A, et al (2005) Modulation of voltage-gated potassium channels in human T lymphocytes by extracellular glutamate. Mol Pharmacol 67: 856–867. 15718225 10.1124/mol.67.3.

[47] Mashkina AP, Tyulina OV, Solovyova TI, Kovalenko EI, Kanevski LM, et al (2007) The excitotoxic effect of NMDA on human lymphocyte immune function. Neurochem Int 51: 356–360. 17543418 10.1016/j.neuint.2007.04.009

[48] Miglio G, Dianzani C, Fallarini S, Fantozzi R, Lombardi G (2007) Stimulation of N-methyl-D-aspartate receptors modulates Jurkat T cell growth and adhesion to fibronectin. Biochem Biophys Res Commun 361: 404–409. 17662248 10.1016/j.bbrc.2007.07.015

[49] Kvaratskhelia E, Maisuradze E, Dabrundashvili NG, Natsvlishvili N, Zhuravliova E, et al (2009) N-methyl-D-aspartate and sigma-ligands change the production of interleukins 8 and 10 in lymphocytes through modulation of the NMDA glutamate receptor. Neuroimmunomodulation 16: 201–207. 19246943 10.1159/000204234

[50] Lindblad SS, Mydel P, Hellvard A, Jonsson IM, Bokarewa MI (2012) The N-methyl-d-aspartic acid receptor antagonist memantine ameliorates and delays the development of arthritis by enhancing regulatory T cells. Neurosignals 20: 61–71. 22134197 10.1159/000329551

